# SERVICE INNOVATION (SOUL PROGRAMME) - Charitable home-based outreach service for treatment of schizophrenia in Larkano, Pakistan: development, implementation and 10 year outcomes

**DOI:** 10.1192/j.eurpsy.2022.290

**Published:** 2022-09-01

**Authors:** S.S. Afghan, B. Junejo, G. Soomro, F. Soomro, R. Wagan, R. Faruqui

**Affiliations:** 1 Dorothy Pattison Hospital, Adult Mental Health, Walsall, United Kingdom; 2 Shaheed Mohtarma Benazir Bhutto Medical University, Psychiatry, Larkana, Pakistan; 3 Petersfield Community Hospital, Community Mental Health Team, Petersfield, United Kingdom; 4 University of Kent, Kent & Medway Medical School, Canterbury, United Kingdom

**Keywords:** Charitable, Developing Country, Outreach service, schizophrénia

## Abstract

**Introduction:**

There is a huge resource gap in mental health service provision & service utilisation in LAMIC including Pakistan. SOUL Programme has been established in the City of Larkana, on charitable donations, which utilises principles of home-based outreach and produces clinical and functional outcomes.

**Objectives:**

SOUL programme focuses on collaborative working with patients & families. The objectives include recognition, treatment, family education & psychosocial support to maximize clinical, functional & occupational outcomes.

**Methods:**

Single cohort intervention (patients recruited on continual basis over time) with innovative service structure and culturally relevant open label intervention design developed with local academic psychiatric unit in Larkano, Pakistan. Training was provided to local mental health professionals on diagnosis, delivering care & use of recognized clinical outcome measures.

**Results:**

We have recruited a cohort of 160 patients on continual basis over time. Our analysis show a higher BPRS and lower GAF ratings for men in comparison to female cohort at the baseline. Our Ten year follow up has demonstrated statistically significant clinical / functional improvement on BPRS, CGI & GAF measures. The mean differences recorded for the individual measures after 12 months were BPRS, CGI-I and GAF and were all statistically significant. Innovative home-based community mental health intervention shows significant improvements in clinical and functional outcomes (with good effect size).

**Conclusions:**

SOUL Programme is a highly effective and cost-efficient intervention model for treatment of schizophrenia in a developing country setting. Our 10 year follow up study confirms the feasibility of this intervention model through close working with families of our patients.

**Disclosure:**

No significant relationships.

